# Survival following abdominal aortic aneurysm repair in North Queensland is not associated with remoteness of place of residence

**DOI:** 10.1371/journal.pone.0241802

**Published:** 2020-11-13

**Authors:** Jonathan Golledge, Aaron Drovandi, Ramesh Velu, Frank Quigley, Joseph Moxon

**Affiliations:** 1 Queensland Research Centre for Peripheral Vascular Disease, College of Medicine and Dentistry, James Cook University, Townsville, Queensland, Australia; 2 The Department of Vascular and Endovascular Surgery, Townsville University Hospital, Townsville, Queensland, Australia; 3 Australian Institute of Tropical Medicine, James Cook University, Townsville, Queensland, Australia; 4 Mater Private Hospital, Townsville, Queensland, Australia; Imperial College London, UNITED KINGDOM

## Abstract

**Objective:**

To assess whether survival and clinical events following elective abdominal aortic aneurysm (AAA) repair were associated with remoteness of residence in North Queensland, Australia.

**Methods:**

This retrospective cohort study included participants undergoing elective AAA repair between February 2002 and April 2020 at two hospitals in Townsville, North Queensland, Australia. Outcomes were all-cause survival and AAA-related events, defined as requirement for repeat AAA repair or AAA-related mortality. Remoteness of participant’s place of residence was assessed by the Modified Monash Model classifications and estimated distance from the participants’ home to the tertiary vascular centre. Cox proportional hazard analysis examined the association of remoteness with outcome.

**Results:**

The study included 526 participants undergoing elective repair by open (n = 204) or endovascular (n = 322) surgery. Fifty-four (10.2%) participants had a place of residence at a remote or very remote location. Participants' were followed for a median of 5.2 (inter-quartile range 2.5–8.3) years, during which time there were 252 (47.9%) deaths. Survival was not associated with either measure of remoteness. Fifty (9.5%) participants had at least one AAA-related event, including 30 (5.7%) that underwent at least one repeat AAA surgery and 23 (4.4%) that had AAA-related mortality. AAA-related events were more common in participants resident in the most remote areas (adjusted hazard ratio 2.83, 95% confidence intervals 1.40, 5.70) but not associated with distance from the participants’ residence to the tertiary vascular centre

**Conclusions:**

The current study found that participants living in more remote locations were more likely to have AAA-related events but had no increased mortality following AAA surgery. The findings emphasize the need for careful follow-up after AAA surgery. Further studies are needed to examine the generalisability of the findings.

## Introduction

Abdominal aortic aneurysm (AAA) is a common cause of death in older adults due to aortic rupture [1]. The only effective treatment to prevent AAA rupture is elective surgical repair by either open or endovascular surgery [2, 3]. A number of prior observational studies have reported that the outcome of AAA repair is worse in hospitals with low volumes and this has led to the centralization of services for AAA repair in some countries, such as the UK and Spain [4–6]. The European Society for Vascular Surgery guidelines recommend that AAA repair should only be considered in centers with a minimum annual case volume of 30 [7]. Centralizing vascular surgery practice has some challenges for consumers and providers, particularly in countries where populations are dispersed over large land areas. The north part of Queensland in Australia has one of the most dispersed populations in the world with a population of around 300,000 dispersed over a land area of more than 200,000 km^2^. Vascular surgery services to this region are provided by one public (Townsville University Hospital) and one private (The Mater Private Hospital Townsville) hospital with a staff of four vascular surgeons [8, 9]. Individuals requiring AAA repair need to attend one of these hospitals, which may require them to travel as far as 900 km. The Northern Queensland region also includes areas with varying availability of primary health services and imaging services with some areas, such as Cardwell, Hughenden, Nelly Bay and Collinsville, recognized as remote or very remote. Living in more remote and distant localities can be associated with challenges in obtaining medical care and attending tertiary hospitals, which is thought to contribute to geographic disparity in the outcomes for many chronic diseases [10, 11].

Complications after open or endovascular AAA repair, such as anastomotic aneurysm, graft infection, endoleak, graft migration, graft occlusion and new aneurysm formation, are well described and therefore current guidelines recommend long-term follow-up with intermittent imaging [7, 12]. Living in remote or distant regions would be expected to pose greater challenges in attending appointments needed to monitor graft failure and thus might be associated with a lower rate of corrective surgery and a higher risk of late AAA rupture. Remoteness is also associated with greater prevalence of chronic diseases which may be association with increased perioperative or long-term mortality [10, 11]. There has however been limited investigation of the association of remoteness of place of residence on long-term outcome after AAA repair. This is an important consideration for whether centralization of services for AAA is appropriate.

The aim of this study was to examine whether outcomes for participants undergoing elective AAA surgery in North Queensland, Australia varied depending on the remoteness of their place of residence, and physical distance to their servicing hospital.

## Materials and methods

### Study design and participants

This investigation was a retrospective study of elective AAA repair at The Townsville University public hospital and The Mater Private Hospital Townsville between February 2002 and April 2020. February 2002 was chosen at the commencement time for this study as this is when the tertiary Vascular and Endovascular Surgery Department in Townsville was established. Participants were eligible for inclusion if they underwent elective surgical repair of an asymptomatic abdominal aortic or abdominal aorto-iliac aneurysm by endovascular or open surgery at either of these hospitals. Emergency repairs of ruptured or symptomatic AAAs were not included. Participants were generally selected for elective repair if they had an asymptomatic AAA measuring ≥50mm in diameter after discussion at a multi-disciplinary meeting. Endovascular AAA repair was favoured for aneurysms that were anatomically suitable including an aneurysm neck length of ≥15mm and suitable access vessels. Open surgery was generally reserved for participants that were deemed fit without severe respiratory disease, evidence of reversible cardiac ischemia on a myocardial perfusion scan or stress test, or heart failure on echocardiography. Ethical approval was granted from the Townsville Hospital and Health Services Ethics Committee (HREC/13/QTHS/125). Public health act approval was obtained for a waiver of consent due to the retrospective design (RD004829). The Strengthening the Reporting of Observational Studies in Epidemiology (STROBE) guidelines for reporting observational studies were followed for the reporting of this study (**S1 Table**) [13].

### Remoteness

Participant’s remoteness was coded according to their suburb of residence using the 2019 Modified Monash Model classifications, which categorises an area based on geographical remoteness and town population size [14]. This was coded as metropolitan or large regional town (categories 1–2), medium regional town (categories 3–4), small regional town (category 5) or remote location (categories 6–7). Physical distance from the hospital where repair was performed, referred to as distance from the tertiary vascular centre, was calculated for each participant, as the estimated driving distance from the participants’ suburb of residence to The Townsville University Hospital or The Mater Private Hospital Townsville, as appropriate, using google maps.

### Definitions of risk factors and medications recorded

Smoking was defined as current smoking (smoking within the last month), ever smoking, or never smoking based on history [15, 16]. Hypertension, diabetes and stroke were defined by a documented past history of diagnosis of these conditions [15, 16]. Ischemic heart disease (IHD) was defined as a documented history of myocardial infarction, angina, or previous treatment of IHD [16]. Participants that self-identified as Aboriginal and Torres Strait Islanders were recorded as such, and other participants were considered as Non-Indigenous Australians. Family history of AAA was defined by at least one first-degree relative with a history of AAA. Relative levels of social advantage and disadvantage was coded based on participant’s residence postcode using the Index of Relative Social Advantage and Disadvantage scores available through the Australian Bureau of Statistics [17]. All prescribed medications were recorded at the time of surgery. Body mass index (BMI) and resting blood pressure were measured as previously described [15, 16].

### Definition and assessment of outcomes

Participants were followed up as part of normal care, usually at 3, 6 and 12 months and then annually following surgery. Outcome data were recorded during clinical reviews and were also obtained from linked hospital admission records using the Queensland hospital admitted patient data collection as previously described [15, 18–20]. These data are regularly audited to minimize inaccuracies [21]. The primary outcome of this study was all-cause survival. The secondary outcome was AAA-related events defined as repeat AAA surgery needed due to failure of aneurysm exclusion or graft occlusion or infection, or aneurysm-related mortality. The latter included any death within 90 days of AAA repair or related to AAA rupture. Causes of death were classified using the International Statistical Classification of Diseases and Related Health Problems (ICD)-10 codes [22].

### Sample size

Since this was a retrospective observational study the sample size was informed by the available participants during the study period rather than as a results of a pre-specified sample size estimate. Prior to analysis the available sample size was considered based on the plan to assess the association between remoteness and survival in an adjusted Cox proportional hazard analysis. Mortality over 5 years was estimated to be approximately 40% based on a prior study in this population [23]. The Cox proportion hazard analyses were planned to include up to 10 covariates of which some, such as Modified Monash Model classifications, had multiple permutations. It was estimated that at least 500 individuals would lead to a well powered analysis considering the requirement to attain at least 10 outcome events per degree of freedom according to Monte-Carlo simulations [24].

### Data analysis

Continuous data were not normally distributed according to the Shapiro Wilk test and were presented as median and inter-quartile range (IQR) and compared between groups using the Mann-Whitney U test. Categorical variables were compared using Pearson’s chi squared test. In order to examine the association of remoteness with survival and AAA-related events, Cox proportional hazard analyses were performed which included adjustment for risk factors that were identified to have bivariate association with the relevant outcome with a p value of < .05. Participants were censored at the time of their first relevant event, or date of last follow-up if no event was experienced. Hazard ratios (HR) and 95% confidence intervals (CI) were presented. Data were analysed using the SPSS v25 (IBM, Armonk, NY) software package. Event incidence curves were generated using the R software package (R Core Team, Vienna, Austria) and statistical analysis performed with log rank test. P values of < .05 were considered significant for all analyses.

## Results

### Characteristics of participants

A total of 526 participants that had endovascular (322; 61.2%) or open surgical (204; 38.8%) AAA repair were included. The proportion of participants having endovascular repair progressively increased throughout the study period (2002 to 2006: 52 of 144, 36.1%; 2007 to 2012: 136 of 214, 63.6%; 2013 to 2020: 134 of 168, 79.8%; p<0.001). Compared to participants having open surgery, those that underwent endovascular AAA repair were significantly older, significantly more likely to have diabetes and had a significantly greater BMI, where measured (**S2 Table**). There was no association between the measures of remoteness or Index of Relative Social Advantage and Disadvantage and method of AAA repair (**S2 Table**).

### Association between rurality and survival

**Fig 1:**
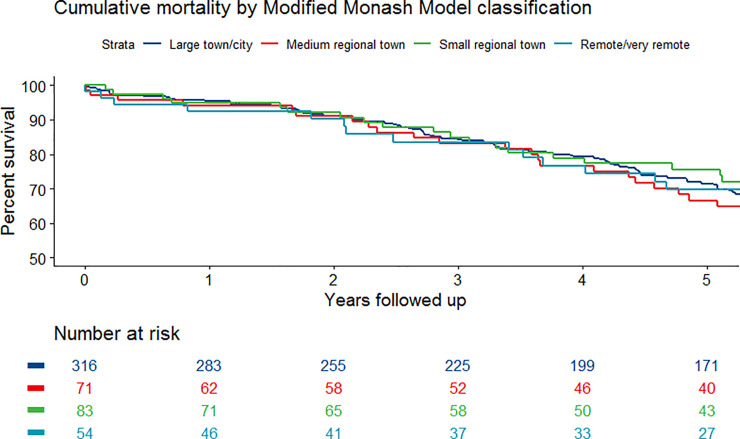
Survival in relation to Modified Monash Model classifications.

Participants were followed for a median of 5.2 (inter-quartile range 2.5–8.3) years. Due to the progressive increased use of endovascular repair in recent years, the available following of participants having endovascular surgery (median 4.6, inter-quartile range 2.1–6.9 years) was less than for participants having open repair (median 7.1, inter-quartile range 3.3–10.5 years; p<0.001). A total of 252 (47.9%) deaths were recorded. Participants that died during follow-up were significantly older and significantly more likely to have IHD and prior stroke (**Table 1**). The participants that died also had a significantly lower BMI and were significantly less likely to be prescribed statins at the time of AAA repair. There was no association between Modified Monash Model classification or distance from the tertiary vascular center and survival (**Tables 1** and **2**and **Fig 1;** log rank test for associated with Modified Monash Model classifications p = .619). Common causes of death included IHD (n = 45; 17.9%), heart failure (n = 16; 6.3%), stroke (n = 15; 6.0%) and cancer (28.2%) (**S3 Table**). In the Cox proportional hazard model, older participants (HR 1.05, 95% CI 1.04–1.07, per year), women (HR 1.41, 95% CI 1.01–1.96) and participants with IHD (HR 1.43, 95% CI 1.11–1.86) or prior stroke (HR 1.74, 95% CI 1.13–2.67) had significantly increased mortality.

**Table 1:** Comparison of risk factors for participants that did or did not die.

Risk factor	Died (N = 252)	Did not die (N = 274)	P value
Open AAA repair	89 (35.3%)	115 (42.0%)	0.118
Endovascular AAA repair	163 (64.7%)	159 (58.0%)	0.118
Age	74.7 (69.4–79.3)	70.6 (65.6–75.0)	**<0.001**
Female	46 (18.3%)	35 (12.8%)	0.082
Aboriginal or Torres Strait Islander	5 (2.0%)	6 (2.2%)	0.869
Family history of AAA	15 (6.0%)	26 (9.5%)	0.131
Current smoker	62 (24.6%)	78 (28.5%)	0.316
Diabetes	45 (17.9%)	46 (16.8%)	0.746
Hypertension	193 (76.6%)	195 (71.2%)	0.158
Ischemic heart disease	143 (56.7%)	124 (45.3%)	**0.008**
Prior stroke	24 (9.5%)	10 (3.6%)	**0.006**
BMI*	26.9 (23.5–30.4)	28.0 (25.1–30.8)	**0.014**
Systolic blood pressure†	134 (120–146)	132 (120–145)	0.414
Diastolic blood pressure‡	75 (68–81)	77 (70–83)	0.250
Anti-platelet drug§	141 (56.0%)	145 (52.9%)	0.424
Statin§	128 (50.8%)	166 (60.6%)	**0.031**
IRSAD§	968 (929–986)	966 (929–986)	0.874
Distance from tertiary vascular centre (km)§	132 (7–386)	112 (10–386)	0.456
Modified Monash classification§			0.734
1–2 (large town/city)	152 (60.3%)	164 (59.9%)	
3–4 (medium regional town)	37 (14.7%)	34 (12.4%)	
5 (small regional town)	36 (14.3%)	47 (17.2%)	
6–7 (remote/very remote)	27 (10.7%)	27 (9.9%)	
Follow-up (years)	4.8 (2.6–7.3)	5.7 (2.2–9.6)	**0.029**

Data are presented as number (percentage), or median (interquartile range). Data only available for 389*, 379†, 378‡, and 524§ participants. IRSAD: Index of Relative Socio-economic Advantage and Disadvantage. Modified Monash Model classification and distance from the tertiary vascular centre were not calculated in two participants visiting from the UK and Papua New Guinea.

**Table 2:** Association of measures of remoteness with mortality following abdominal aortic aneurysm repair.

Remoteness measure	Death		AAA-related events	
	Unadjusted	Adjusted‡	Unadjusted	Adjusted§
Modified Monash classification*				
3–4 (medium regional town)	0.92	0.98	0.82	0.86
	(0.64–1.33)	(0.68–1.42)	(0.34–1.97)	(0.35–2.08)
5 (small regional town)	0.91	0.91	0.52	0.55
	(0.63–1.31)	(0.63–1.31)	(0.18–1.49)	(0.19–1.56)
6–7 (remote/very remote)	1.24	1.37	2.67	2.83
	(0.82–1.87)	(0.91–2.07)	(1.33–5.36)	(1.40–5.70)
Distance from tertiary vascular centre†	0.91	0.96	1.03	1.04
	(0.81–1.02)	(0.85–1.08)	(0.82–1.31)	(0.82–1.32)

This analysis does not include two participants visiting from the UK and Papua New Guinea.

*Reference category is 1–2 (large town/city).

†per 300 km (approximate standard deviation).

‡Adjusted for age, ischemic heart disease, prior stroke and statin prescription. Body mass index not included as missing in 133 participants.

**§**Adjusted for prior stroke.

### Association between rurality and AAA-related events

**Fig 2:**
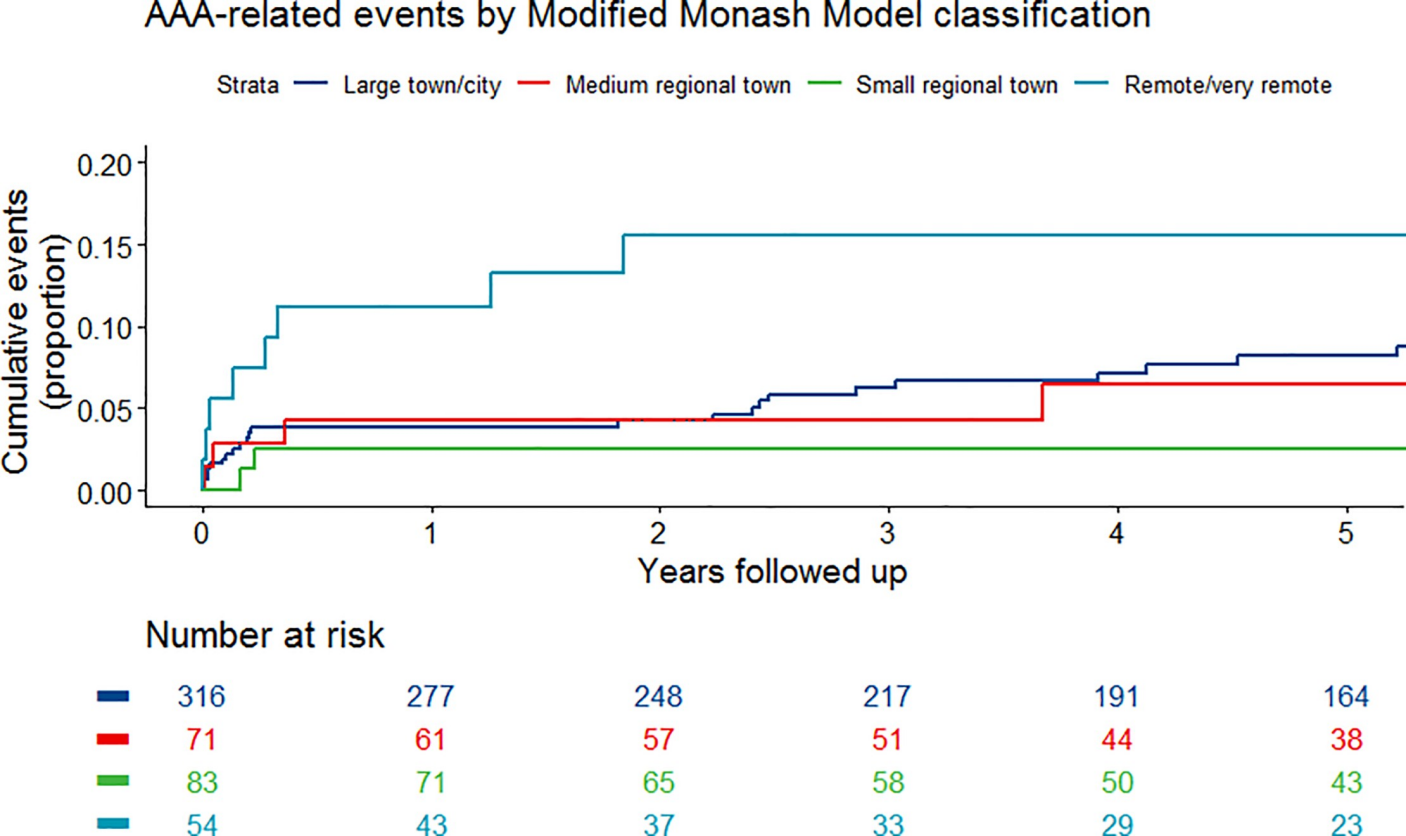
AAA-related events in relation to Modified Monash Model classifications.

Fifty (9.5%) participants had at least one AAA-related event, including 30 (5.7%) participants that underwent at least one repeat AAA surgery (7 had two repeat operations and 3 had three repeat surgeries) and 23 (4.4%) that had AAA-related mortality due to death within 90 days of elective AAA repair (14), graft infection (4) or late AAA rupture (5). Three participants had both repeat AAA surgery and later died within 90 days of the original repair (2) or due to later graft infection (1). AAA-related events were significantly more common in participants with prior stroke and those with a place of residence that was in a location that was remote or very remote (**Table 3**). Eleven of the 54 (22.0%) participants resident in remote locations (Modified Monash Model classifications 6 or 7) had an AAA-related event compared 29 of the 316 (9.2%) in participants resident in a metropolitan or large regional town (Modified Monash Model classifications 1 or 2). Amongst participants resident in remote locations, two died within 90 days of surgery, eight required repeat AAA repairs (7 participants had one repair and one participant 3 repairs) and one died from late AAA rupture following failed endovascular repair. Cox proportional hazard analyses showed that participants’ resident in remote locations had a higher risk of AAA-related events both before (HR 2.67, 95% CI 1.33–5.36) and after (HR 2.83, 95% CI 1.40–5.70) adjusting for other risk factors (**Table 2**). Distance from the tertiary vascular centre was not associated with AAA-related events (**Tables 2** and **3**). AAA-related events were significantly associated with Modified Monash Model classifications (Log rank p = .006; **Fig 2**).

**Table 3:** Comparison of risk factors for participants that did or did not have an abdominal aortic- related event.

Risk factor	Abdominal aortic aneurysm-related events		P value
	**Yes (N = 50)**	**No (N = 476)**	
Open AAA repair	14 (28.0%)	190 (39.9%)	0.100
Endovascular AAA repair	36 (72.0%)	286 (60.1%)	0.100
Age	74.8 (69.4–78.0)	72.5 (66.7–77.3)	0.082
Female	7 (14.0%)	74 (15.5%)	0.773
Aboriginal or Torres Strait Islander	1 (2.0%)	10 (2.1%)	0.962
Family history of AAA	6 (12.0%)	35 (7.4%)	0.244
Current smoker	12 (24.0%)	128 (26.9%)	0.660
Diabetes	8 (16.0%)	83 (17.4%)	0.798
Hypertension	41 (82.0%)	347 (72.9%)	0.164
Ischemic heart disease	31 (62.0%)	236 (49.6%)	0.095
Prior stroke	8 (16.0%)	26 (5.5%)	**0.004**
BMI*	27.2 (24.7–31.3)	27.4 (24.6–30.6)	0.850
Systolic blood pressure†	140 (126–151)	131 (120–145)	0.065
Diastolic blood pressure‡	79 (70–83)	75 (69–82)	0.348
Anti-platelet drug§	29 (58.0%)	257 (54.2%)	0.610
Statin§	33 (66.0%)	261 (55.1%)	0.138
IRSAD§	966 (928–985)	967 (929–986)	0.743
Distance from the tertiary vascular centre	103 (7–387)	132 (8–386) §	0.841
Modified Monash classification			**0.022**
1–2	29 (58.0%)	287 (60.5%)§	
3–4	6 (12.0%)	65 (13.7%)§	
5	4 (8.0%)	79 (16.7%)§	
6–7	11 (22.0%)	43 (9.1%)§	
Follow-up	5.3 (0.5–7.5)	5.2 (2.7–8.5)	0.233

Data are presented as number (percentage), or median (interquartile range). Data only available for 389*, 379†, 378‡, and 524§ participants. IRSAD: Index of Relative Socio-economic Advantage and Disadvantage. Modified Monash Model classification and distance from the tertiary vascular centre were not calculated for two participants visiting from the UK and Papua New Guinea.

## Discussion

Elective aortic surgery is offered as a treatment to prevent AAA rupture in order to increase survival in individuals with large AAAs. This study is the first to assess the impact of geographic variation on the outcome following elective AAA repair in Australia. The analysis was focused on the large geographic region of Northern Queensland. The main finding was that there was no relationship between survival and measures of remoteness, but that participants resident in the most remote locations were more likely to have an AAA-related event. The findings suggest that while it is feasible to offer an elective AAA repair service to a population dispersed over a wide geographic region without disparity in mortality, careful follow-up after repair is important in order to identify the need for repeat AAA surgery.

Late complications, such as endoleak, anastomotic aneurysm, graft migration, graft occlusion and new aortic aneurysm formation, have been reported following open or endovascular AAA repair [2, 25, 26]. Current guidelines recommend repeat aortic imaging and follow-up after AAA surgery, particularly endovascular repair [7, 12]. In the current study it was noted that endovascular repair has become the main method of repairing asymptomatic AAAs in North Queensland. 30 (5.7%) participants required at least one repeat AAA surgery and five (1.0%) had late AAA ruptures. Attending follow-up might be expected to be more difficult for individuals living in more remote locations [27]. AAA-related events were more common in participants resident in remote locations but only one of these was related to AAA rupture. This participant had failed endovascular AAA repair and was deemed unfit for open AAA repair thus could not have been avoided by more intense follow-up. The majority of AAA-related events (8 of 11) amongst participants living in remote locations were related to the requirement for repeat AAA surgery. While no information was collected on attendance for follow-up, these findings suggests that living in a remote locality is not a risk factor for late rupture after endovascular repair. Thus, remoteness does not appear to be a reason to recommend against endovascular AAA repair as long as it is feasible to attend follow-up in order to detect the need for secondary AAA repair.

In some countries, such as the USA, individuals living in more rural localities attend smaller hospitals for AAA repair [28]. A number of prior systematic reviews have highlighted potential concerns that this approach may result in higher mortality rates due to inexperienced surgical teams performing AAA repair [5, 6, 29]. In North Queensland, Australia, AAA repair is performed by Royal Australasian College of Surgeons accredited vascular surgeons at one public and private hospital. This means populations dispersed over a large land mass need to attend these tertiary facilities. The advantage of this centralized approach is that surgeons, anesthetists, nurses and support staff gain increased experience of AAA repair procedures and care pathways. Such a centralized approach is favored in a number of other countries such as the UK and has been reported to reduce perioperative mortality [30]. In contrast to this intense interest in the relationship between hospital and surgeon volumes and the outcome of AAA repair, there has been relatively little study of the relationship between place of residence and the outcome of elective AAA repair. No prior studies of the relationship between remoteness of place of residence and long-term outcome of elective AAA repair were identified. A previous USA study reported that individuals living in more rural locations had reduced perioperative mortality following elective AAA repair [20]. The relationship between remoteness and outcome of ruptured AAA has been studied in Australia and Norway. A report from Tasmania suggested that living in a more rural locations was not associated with increased mortality from ruptured AAA [31]. Geographic disparity in the outcome of ruptured AAA has been reported in Norway, although the exact relationship between measures of remoteness was not studied [32]. In contrast, a previous population study in New South Wales reported no relationship between remoteness of residence and community mortality following AAA rupture [33]. It is likely however that some sudden deaths due to AAA rupture are not recorded as an AAA-related mortality.

The current study has a number of strengths and limitations. Strengths include the large number of participants recruited from a wide geographic region serviced by one public and one private tertiary hospital with a consistent treating team over an extended period. Limitations of the study include the retrospective design, that the study may have been underpowered since only 54 participants were from remote or very remote locations and the lack of information about post-operative imaging. Post-mortem rates in Australia are very low, and while causes of death were obtained from death records it is possible that some AAA-related mortalities were not correctly coded. Furthermore, the findings may not be generalizable to other locations in Australia or internationally where health systems and population density are different. Finally, it is possible that differences in primary care services or referral patterns in different geographic regions may have contributed to the findings.

In summary, the current study found that within North Queensland, Australia, survival following elective AAA surgery was unrelated to place of residence. AAA-related events were more common in participants located in remote regions highlighting the need for careful follow-up.

## Supporting information

S1 Table Completed STROBE statement checklist. (DOCX)

S2 Table Comparison of risk factors of participants undergoing open and endovascular abdominal aortic aneurysm repair. Data are presented as number (percentage), or median (interquartile range). Data only available for 389*, 379†, 378‡, and 524§ participants. IRSAD: Index of Relative Socio-economic Advantage and Disadvantage. Modified Monash Model classification and distance from the tertiary vascular centre were not calculated for two participants visiting from the UK and Papua New Guinea (one in the endovascular group and one in the open group).
(DOCX)

S3 Table Causes of death. (DOCX)
