# Supplementary material for: Survival following abdominal aortic aneurysm repair in North Queensland is not associated with remoteness of place of residence
Source: PLoS One. 2020 Nov 13;15(11):e0241802. doi: 10.1371/journal.pone.0241802 (PMC7665769; doi:10.1371/journal.pone.0241802)
Supplement: S2 Table — Data are presented as number (percentage), or median (interquartile range). Data only available for 389*, 379†, 378‡, and 524§ participants. IRSAD: Index of Relative Socio-economic Advantage and Disadvantage. Modified Monash Model classification and distance from the tertiary vascular centre were not calculated for two participants visiting from the UK and Papua New Guinea (one in the endovascular group and one in the open group). (DOCX) [file pone.0241802.s002.docx]

**S2 Table. Comparison of risk factors of participants undergoing open and endovascular abdominal aortic aneurysm repair**

| **Risk factor** | **Surgical repair method** | | **P value** |
| --- | --- | --- | --- |
|  | **Endovascular (N=322)** | **Open (N=204)** |  |
| Age | 73.5 (68.1-78.8) | 71.0 (65.2-75.5) | **<.001** |
| Female | 50 (15.5%) | 31 (15.2%) | .918 |
| Aboriginal or Torres Strait Islander | 7 (2.2%) | 4 (2.0%) | .868 |
| Family history of AAA | 26 (8.1%) | 15 (7.4%) | .764 |
| Current smoker | 81 (25.2%) | 59 (28.9%) | .341 |
| Diabetes | 67 (20.8%) | 24 (11.8%) | **.008** |
| Hypertension | 238 (73.9%) | 150 (73.5%) | .922 |
| Ischemic heart disease | 172 (53.4%) | 95 (46.6%) | .126 |
| Prior stroke | 22 (6.8%) | 12 (5.9%) | .666 |
| BMI* | 27.5 (25.0-31.4) | 26.9 (24.0-29.5) | **.020** |
| Systolic blood pressure† | 131 (120-146) | 135 (121-144) | .375 |
| Diastolic blood pressure‡ | 75 (68-82) | 78 (70-82) | .067 |
| Anti-platelet drug§ | 174 (54.4%) | 112 (54.9%) | .906 |
| Statin§ | 170 (53.1%) | 124 (60.8%) | .085 |
| IRSAD§ | 964 (929-986) | 968 (929-986) | .842 |
| Distance from tertiary vascular centre (km)§ | 118 (8-386) | 137 (9-386) | .471 |
| Modified Monash classification§ |  |  | .405 |
| 1-2 (large towns/city) | 197 (61.4%) | 119 (58.6%) |  |
| 3-4 (medium regional town) | 41 (12.8%) | 30 (14.8%) |  |
| 5 (small regional town) | 46 (14.3%) | 37 (18.2%) |  |
| 6-7 (remote/very remote) | 37 (11.5%) | 17 (8.4%) |  |

Data are presented as number (percentage), or median (interquartile range). Data only available for 389*, 379†, 378‡, and 524§ participants. IRSAD: Index of Relative Socio-economic Advantage and Disadvantage. Modified Monash Model classification and distance from the tertiary vascular centre were not calculated for two participants visiting from the UK and Papua New Guinea (one in the endovascular group and one in the open group).
