# Supplementary material for: Survival following abdominal aortic aneurysm repair in North Queensland is not associated with remoteness of place of residence
Source: PLoS One. 2020 Nov 13;15(11):e0241802. doi: 10.1371/journal.pone.0241802 (PMC7665769; doi:10.1371/journal.pone.0241802)
Supplement: S3 Table — (DOCX) [file pone.0241802.s003.docx]

**S3 Table. Causes of death**

| **Cause of death** | **Number** | **Percentage** |
| --- | --- | --- |
| AAA-related | 23 | 9.1% |
| **Other cardiovascular disease** | | |
| Other aneurysm or peripheral artery disease | 9 | 3.6% |
| Coronary heart disease | 45 | 17.9% |
| Heart failure | 16 | 6.3% |
| Stroke | 15 | 6.0% |
| Ischemic bowel | 5 | 2.0% |
| Cancer | 71 | 28.2% |
| Bladder | 4 |  |
| Breast | 1 |  |
| Brain | 1 |  |
| Colon | 7 |  |
| Gastric | 1 |  |
| Laryngeal | 1 |  |
| Leukaemia | 3 |  |
| Lung | 16 |  |
| Lymphoma | 1 |  |
| Multiple myeloma | 2 |  |
| Oesophageal | 3 |  |
| Ovarian | 1 |  |
| Pancreas | 3 |  |
| Prostate | 13 |  |
| Renal cell carcinoma | 1 |  |
| Skin | 2 |  |
| Other cancer | 11 |  |
| **Other causes** | | |
| Airway disease or pneumonia | 35 | 13.9% |
| Chronic renal disease | 5 | 2.0% |
| Sepsis | 8 | 3.2% |
| Dementia | 7 | 2.8% |
| Parkinson’s disease | 1 | 0.4% |
| Fracture or trauma | 6 | 2.4% |
| Pancreatitis | 1 | 0.4% |
| Peptic ulcer | 2 | 0.8% |
| Pulmonary embolism | 1 | 0.4% |
| Related to other surgical procedure | 1 | 0.4% |
| Unknown | 1 | 0.4% |
